# High Serum Levels of IL-6 Are Associated with Suicide Attempt but Not with High Lethality Suicide Attempts: A Preliminary Case–Control Study

**DOI:** 10.3390/ijerph192214735

**Published:** 2022-11-09

**Authors:** Rosa Giannina Castillo-Avila, Alma Delia Genis-Mendoza, Isela Esther Juárez-Rojop, María Lilia López-Narváez, Diana María Dionisio-García, Germán Alberto Nolasco-Rosales, Miguel Ángel Ramos-Méndez, Yazmín Hernández-Díaz, Carlos Alfonso Tovilla-Zárate, Thelma Beatriz González-Castro, Humberto Nicolini

**Affiliations:** 1 División Académica de Ciencias de la Salud, Universidad Juárez Autónoma de Tabasco, Villahermosa 86100, Mexico; 2 Laboratorio de Genómica de Enfermedades Psiquiátricas y Neurodegenerativas, Instituto Nacional de Medicina Genómica, Ciudad de Mexico 14610, Mexico; 3 Hospital Chiapas Nos Une “Dr. Gilberto Gómez Maza”, Secretaría de Salud, Tuxtla Gutierrez 29045, Mexico; 4 División Académica Multidisciplinaria de Jalpa de Méndez, Universidad Juárez Autónoma de Tabasco, Jalpa de Mendez 86040, Mexico; 5 División Académica Multidisciplinaria de Comalcalco, Universidad Juárez Autónoma de Tabasco, Comalcalco 86040, Mexico

**Keywords:** suicide attempt, IL-6, serum, polymorphism, *IL6R* gene, lethality

## Abstract

Suicide attempts are an emerging health problem around the world. Increased levels of IL-6 have been associated with suicidal behavior. Therefore, the aims of this study were to evaluate the serum levels of IL-6 in individuals with suicide attempts and a comparison group and to associate the IL-6 levels with the lethality of the suicide attempt. Additionally, we associated the rs2228145 polymorphism of the *IL6R* gene with suicide attempts or with the IL-6 serum levels. Suicide attempts and their lethality were evaluated using the Columbia Suicide Severity Rating Scale. The serum concentrations of IL-6 were measured by the ELISA technique in individuals with suicide attempts and then compared to a control group. The rs2228145 polymorphism of the *IL6R* gene was analyzed by real-time polymerase chain reaction. We found elevated serum levels of IL-6 in the suicide attempt group when compared to the control group (F = 10.37, *p* = 0.002). However, we found no differences of the IL-6 levels between high and low lethality. The *IL6R* gene polymorphism rs2479409 was not associated with suicide attempts. Our data suggest that IL-6 serum is increased in individuals with suicide attempts.

## 1. Introduction

Suicide attempts are defined as an act in which an individual harms himself or herself with the intent to die, but he or she survives [1]. This represents an important global public health problem, with a subgroup of people at high risk of dying [2].

Each year, approximately 700,000 people die by suicide [3]. It is estimated that, for every completed suicide, there are around 20 suicide attempts worldwide; nonetheless, some reports indicate that some countries have higher rates of suicide fatalities, as 8.5–13% of all suicide attempts are fatal [4,5,6].

In this sense, the personal history of suicide attempts and family history of suicides have been described as two of the main established and relevant risk factors for subsequent suicide attempts and suicide deaths [7], hence the importance of studying suicide attempts.

Additionally, the lethality of previous suicide attempts can predict and help to understand the risk of a future suicide in patients hospitalized for suicidal behavior and ideation [8]. Therefore, it is important to consider the degree of lethality to identify candidate biomarkers in suicide research, as well as genetic variants of reported genes, in order to elucidate the mechanisms involved in suicide attempts.

Various studies have contemplated the idea that lethality may be associated with some markers and the levels of interleukins. Nonetheless, no studies have evaluated the degree of lethality of suicide attempts and the level of IL-6 in the serum; therefore, this variable of interest opens up a possibility of study to better understand the pathophysiology of suicide attempts.

It is important to implement adequate prevention strategies with the aim to achieve early care and to improve the quality of life of individuals at risk. Prevention should be focused on reducing mortality by including universal interventions that involve pharmacological treatments; public health initiatives and social initiatives [9,10]; and the research of risk factors (for example, the degree of lethality), as well as a timely participation of an interdisciplinary team (doctor, psychiatrist, psychologist, and nurse) [11,12].

It has been described that the multiple interactions of biological, social, personal, environmental, cultural, psychological, and genetic factors favor the transition from ¿uicidal ideation to suicide attempts [2,11,13]. Some factors that influence suicide attempts include inflammatory processes in which interleukins could participate in the development of the entity [14]—in particular, IL-6.

Interleukin-6 (IL-6) belongs to the family of proinflammatory cytokines, and it induces the expression of various proteins involved in the processes of acute inflammation; it also has an important role in cell differentiation and proliferation in humans [15]. IL-6 triggers the responses of some cell types and also acts on target cells through the interleukin-6 receptor (IL-6R), which together induce dimerization of a second receptor subunit, gp130 [16].

It has been described that IL-6 plays an important role in the physiological homeostasis of neural tissue and in the pathogenesis of inflammatory disorders, including diseases where there are profound neuropathological changes; for instance, in Alzheimer’s, Parkinson’s, and multiple sclerosis, an increased expression of this cytokine has been observed in the brain [17]. Indeed, studies suggest that elevated levels of this cytokine may affect neuronal plasticity, neurogenesis, and neurotransmission by modulating behavioral output from the brain; however, the exact mechanism has not been elucidated [18,19,20].

IL-6 has also been related to mental disorders such as anxiety, depression, and suicidal behavior [21]. Some studies have reported that the plasma levels of IL-6 are decreased, while the levels of IL-6 in the serum and cerebrospinal fluid are increased in individuals with suicidal behavior [9]. It has been described that recent and old suicidal behavior could be associated with increased levels of IL-6 [22]; for instance, increased levels of this cytokine in children with suicidal tendencies prior to pharmacological treatment were associated with subsequent suicidal tendencies [23]. Although IL-6 is one of the most studied cytokines, there are discrepancies in the results, and to date, its role in suicidal behavior is not fully understood. Thus, more scientific studies are required.

On the other hand, various polymorphisms of the *IL6R* gene (interleukin 6 receptor) [24] have been associated with mental diseases. The rs2228145 polymorphism (*IL6R* A > C; Asp358Ala) of the *IL6R* gene [25] decreases the inflammatory process by affecting *IL6R* signaling [26]. Nonetheless, the participation of this genetic variant is not fully understood in suicide attempts [24].

Therefore, it is necessary to perform studies that include genetic variants of those genes of interest and inflammatory cytokines such as IL-6 that are involved in the pathogenesis of mental illnesses [27] in order to elucidate the role of these molecules and their possible participation in the pathways or mechanisms that lead to suicide attempts. In this sense, the objectives of our study were, first, to determine if suicide attempts are associated with elevated levels of IL-6 in the serum, second, to evaluate the differences in the IL-6 levels related to the lethality of the suicide attempts and, third, to analyze the association of the rs2228145 polymorphism of the *IL6R* gene in individuals with suicide attempts.

## 2. Materials and Methods

### 2.1. Study Design

This is an observational and cross-sectional study performed in Tabasco, Mexico. This study was performed from January to December 2020.

### 2.2. Participants

The participants were recruited from three hospitals: (a) the High Specialization Regional Hospital “Dr. Gustavo A. Rovirosa Pérez” (Villahermosa City), (b) the “High Specialty Regional Hospital of Mental Health” (Villahermosa City), and (c) the General Hospital of Comalcalco “Dr. Desiderio G. Rosado Carbajal” (Comalcalco City).

The total sample included 84 participants and was divided into two study groups: (a) the suicide attempt group and (b) the comparison group.

### 2.3. Inclusion Criteria for the Suicide Attempt Group

The suicide attempt group (*n* = 18 participants) included: (a) individuals ≥ 18 years of age, (b) of both sexes, (c) diagnosed with suicide attempt by a specialized psychiatrist, (d) the suicidal behavior was evaluated using the Columbia Suicide Severity Rating Scale (C-SSRS), and (e) individuals who arrived at the emergency services due to a suicide attempt.

### 2.4. Inclusion Criteria for the Comparison Group

The comparison group (*n* = 66 participants) included: (a) people without psychiatric illness evaluated by a psychiatrist, (b) without a history of suicide attempts, (c) volunteers, (d) both sexes, and (e) over 18 years of age. Individuals included in the comparison group were recruited from the blood banks of the hospitals previously mentioned when they attended as voluntary blood donors.

### 2.5. Exclusion Criteria for Both Study Groups

People who did not agree to participate in the study or did not agree to sign the informed consent and underage individuals were excluded (≤18 years).

### 2.6. Sociodemographic Characteristics

Sociodemographic information was obtained through face-to-face interviews. We gathered the following data: age, sex, marital status, occupation, years of schooling, and alcohol and cigarette consumption. In addition, the weight (kg) and height (m) were measured. Subsequently, the body mass index (BMI) was calculated using the formula Kg/m^2^.

### 2.7. Assessment of Suicide Attempted and Lethality

The Columbia Suicide Severity Rating Scale (C-SSRS) is a structured and reliable questionnaire that assesses suicidal behavior and suicidal ideation in research settings and in daily clinical practice [28]. In addition, this instrument has been validated in the Spanish language [29]. This scale has 4 subscales: (a) severity of ideation (severity subscale), (b) intensity of ideation subscale (intensity subscale), (c) behavior subscale, and (d) lethality subscale [30].

We used the specific lethality subscale included in the fourth section of the C-SSRS to assess the lethality of suicide attempts. There are other scales to measure suicide attempts (such as the Suicide Intention Scale or Patient Health Questionnaires) [31,32], and we used the Columbia-Suicide Severity Rating Scale (C-SSRS) as the instrument recommended by the US Food and Drug Administration for clinical trials, which has also been used by the Centers for Disease Control and Prevention to stratify and define suicidal behavior [28]. We also chose the C-SSRS to be consistent with previous studies that have evaluated suicidal ideation and intent [33,34].

### 2.8. Interleukins Levels

The levels of interleukin 6 were measured by the ELISA technique (Enzyme-Linked Immunosorbent Assay) using the IL-6 Human ELISA Kit from Invitrogen (96 Tests, Cat. No.-BMS213-2, Invitrogen) following the manufacturers’ protocols.

### 2.9. Genotyping

We extracted high-quality DNA from white blood cells following the modified Lahiri protocol [35]. Then, we analyzed the rs2228145 polymorphism (assay C__16170664_10) with context sequence (VIC/FAM): AATTTTTTTTTTAACCTAGTGCAAG[C/A]TTCTTCTTCAGTACCACTGCCCACA using exonuclease TaqMan genotyping assays on a QuantStudio^TM^ 5 Real-Time PCR System equipment following the manufacturer’s instructions (Thermo Fisher Scientific).

### 2.10. Ethical Statement

The study was conducted in accordance with the Declaration of Helsinki. The study was also approved by the ethics committee of the High Specialty Regional Hospital of Mental Health with registration number HRAESM/DG/UWI/351/2022. All the participants were informed about the type of study, objectives, and procedures. Informed consent was obtained from every individual, and their participation was voluntary.

Due to the importance and sensitivity of the individuals under study, we explained in detail the confidentiality of the data and purposes of the research. Additionally, our working group fostered a respectful and safe environment, avoiding discrimination so that the participants could share their points of view and experiences, considering the psychological or emotional impact that remembering an event could imply.

Finally, the people identified as being at risk were directed to a specialized service to receive the appropriate support.

### 2.11. Statistical Analysis

Analyses were performed using IBM SPSS Statistics 20.0 (IBM, Armonk, NY, USA). Data are expressed as the frequencies and percentages for categorical variables and means ± SD for continuous variables. We also used the chi-square (*x*^2^) or *t*-test for comparing characteristics between individuals with suicide attempts and the comparison group.

We determined the association of the IL-6 levels and suicide attempt lethality. For this analysis, we considered the IL-6 level as the independent variable. We performed an analysis adjusted for age and sex. *p* < 0.05 was considered statistically significant. In multiple comparisons, the level of statistical significance was set at *p* < 0.016 (*P*_correction_ = 0.05/3) using Bonferroni correction.

The genotypic frequencies were tested for Hardy–Weinberg equilibrium using chi-square (*x*^2^). The differences in genotype and allele frequencies of the rs2228145 polymorphism of the *IL-6* gene between the suicide attempt group and the comparison group was compared by chi-square (*x*^2^). For the calculations of the power, we use Quanto software version 1.2.4 (University of Southern California, Los Angeles, CA, USA) [36] with the minor allele frequency = 0.45, *n* = 84. Dominant model, effect size = 1.0. We obtained a power of 0.05.

## 3. Results

### 3.1. Sociodemographic Characteristics

The demographic characteristics of the study population are shown in Table 1. In the overall population, the majority of participants were men, *n* = 49 (58.3%), and the marital status was married, *n* = 42 (50%). Most of the participants were homemakers, *n* = 26 (31%), and had studied 9.79 ± 4.17 years. The median age was 42.19 ± 12.08. Regarding the clinical characteristics, the majority did not consume alcohol, *n* = 47 (56%), or smoke cigarettes, *n* = 74 (88.1%). As for anthropometric measurements, the average of weight was 80.01 ± 16.34 Kg, height 163.14 ± 9.78 cm, and the body mass index 29.93 ± 4.90 kg/m^2^.

In the comparison group, there were more men, *n* = 45 (68.2%). The majority were married, *n* = 34 (51.5%), and full-time employed *n* = 21 (31.8%). The median age was 43.71 ± 11.83, and they studied an average of 9.34 ± 3.83 years. Regarding the clinical characteristics, the majority did not consume alcohol, *n* = 36 (54.5%).

The suicide attempt group included more women, *n* = 14 (77.8%). The majority were single, *n* = 9 (50%), and homemakers, *n* = 7 (38.9%). The average age was 36.63 ± 11.64 years, and the schooling years were 11.16 ± 5.12. The majority did not consume alcohol, *n* = 11 (61.1%), or smoke cigarettes, *n* = 15 (83.3%). The mean weight was 74.72 ± 14.07 kg, height 164.37 ± 10.12 m, and body mass index 29.61 ± 5.03 kg/m^2^.

### 3.2. IL-6 Levels in Suicide Attempt and Comparison Groups

**Figure 1:**
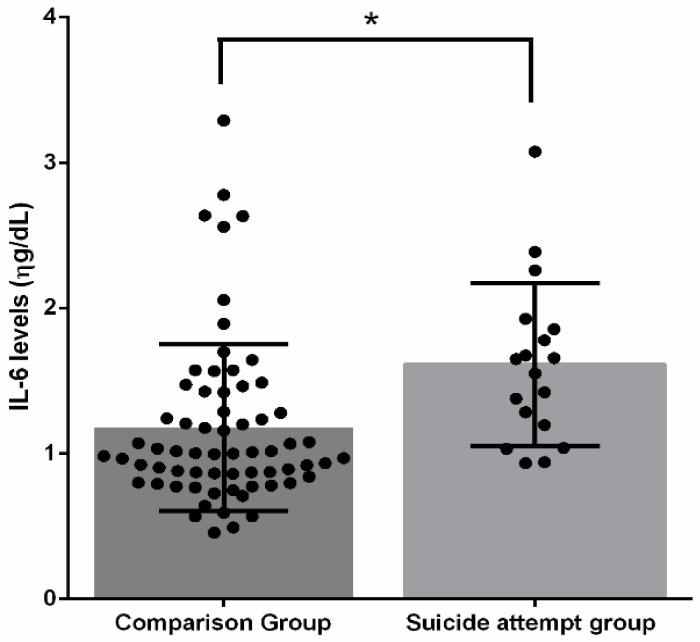
IL-6 levels in suicide attempts and comparison groups. * Statistical significance; ● Participants.

The levels of IL-6 in the serum of individuals with suicide attempts and the comparison group are shown in Figure 1. The suicide attempt group showed statistically significant increased levels of IL-6 (1.61 ± 0.55) when compared to the control group (1.17 ± 0.57). (F = 10.37, *p* = 0.002).

### 3.3. IL-6 Levels and the Lethality of Suicide Attempt

We measured the lethality of the attempted suicides and observed a lethality 0 in eleven participants and a lethality 2 in seven individuals with suicide attempts.

Additionally, the mean IL-6 levels in the serum per subgroup were: 1.56 ± 0.63 for lethality 0, 1.68 ± 0.43 for lethality 2, and 1.17 ± 0.57 for the comparison group.

Subsequently, to determine the association between the IL-6 levels and lethality, we performed comparisons adjusted for age and sex. We did not observe differences between these subgroups.

**Figure 2:**
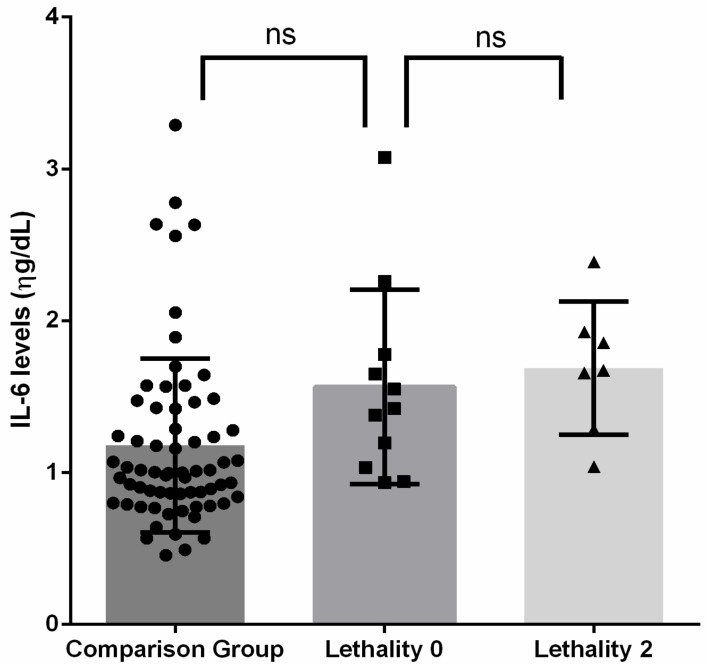
IL-6 levels in lethality of suicide attempts. ns: no statistical significance. The square triangle represents the comparison between groups.

Finally, we performed multiple comparisons with a post-hoc analysis between lethality 0, lethality 2, and the comparison group; however, we did not observe statistically significant differences (F = 1.928, *p* = 0.152) (Figure 2).

### 3.4. Genotype and Allele Distributions of rs2228145 Polymorphism of the *IL6R* Gene

No deviation from the Hardy–Weinberg equilibrium was found in the suicide attempts or comparison groups (*p* > 0.05). In Table 2, we show the genotype and allele distributions of the *IL6R* rs2228145 in the suicide attempts and comparison groups. No statistically significant differences were observed by genotype or allele distributions. Finally, when we evaluated the association of the polymorphism rs2228145 of the *IL6R* gene with the levels of IL-6 in the suicide attempts and comparison groups in the multivariate analysis, no statistically significant difference was observed (F = 0.17, *p* = 0.35).

## 4. Discussion

Interleukin 6 has antiapoptotic and anti-inflammatory functions and various contrary effects that possibly depend on its local concentration, which itself varies, depending on the biological circumstances [37]. The deregulation or increased expression of the *IL6R* gene contributes significantly to the development and pathogenesis of various diseases in humans [38].

IL-6 has been linked with numerous biological functions in the central nervous system (CNS), such as stress response, neurogenesis, and other functions related to the control of behavioral traits, including suicidal behavior [26,39,40]. Therefore, our primary aim was to evaluate the serum levels of the IL-6 levels in the suicide attempts and comparison groups. Additionally, we addressed if the serum IL-6 levels are increased according to the level of lethality. Finally, we associated the polymorphism rs2228145 of the *IL6R* gene with suicide attempted and with the level of IL-6.

First, we observed that the suicide attempt group showed statistically significant higher levels of IL-6 in the serum than the comparison group. This result is in line with previous findings [22]. A meta-analysis reported that the serum IL-6 levels are elevated in individuals with suicidal behavior [9]. Specifically, sustained elevations of IL-6 in the CNS are a key contributor of depressive symptoms and resistance to medications. In fact, excessive IL-6 levels have been related to a poor prognosis and a worse outcome in these patients [39,41,42]. One possible explanation is that the high IL-6 concentrations in the brain block classical or trans-signaling pathways related to the release of neurotransmitters that regulate behavior [40]. Regarding suicidal behavior, evidence shows that elevated IL-6 concentrations are determinant in this conduct [40,41]. Therefore, we could assume that an adequate balance of pro and anti-inflammatory cytokines is essential for the brain homeostasis.

Second, we measured the lethality of attempted suicide using the C-SSRS. Previous studies have used the C-SSRS to assess the lethality in suicide attempts [43]. High lethality in suicide attempts can be considered a close indicator of future completed suicide, and it is essential to study its neurobiology to elucidate the pathophysiology of suicide [44]. We recognize the importance of determining the lethality in suicide attempts in order to identify and refer patients to specialized care areas early and in a timely manner [45]. In this study, we did not observe differences between the degree of lethality in individuals with suicide attempts. One previous study reported an association between suicide attempt lethality and other biomarkers suggesting the C-reactive protein as a peripheral biomarker; however, measuring the IL-6 levels was not within the study objectives [6]. Similarly, the total cholesterol, high LDL-c, HDL-c, and triglycerides have been associated with the lethality of suicide attempts [46]. In the same way, the ratio of platelets to lymphocytes and the mean platelet volume have been associated with high lethality in suicide attempts [47]. Our results, however, suggest that the IL-6 levels might not be modified according to the lethality of suicidal behavior in individuals at risk. It is necessary to replicate the study in other populations, including larger samples of participants.

Third, we analyzed the association between the rs2228145 polymorphism of the *IL6R* gene and individuals with suicide attempts, for the minor allele of the rs2228145 polymorphism (Ala) has been associated with elevated IL-6 levels in the serum and circulating/plasma levels in individuals with schizophrenia [48]. Then, we considered it important to evaluate the genotypic distribution of the *IL6R* gene in suicide attempts. This gene is known to regulate IL-6 bioactivity; one of the most implicated variants with a direct effect on the expression of the *IL6R* gene is the Asp358Ala, rs2228145 A > C (formerly known as rs8192284) [26,49]. Namely, the minor 358Ala allele (C allele) has been associated with a reduction of IL-6R on the surfaces of cells, with a decreased inflammatory activity as the consequence [26,50]. Nevertheless, our analysis did not reveal a statistical association. This could be due to the small number participants included in our study. We could hypothesize that the Asp358Ala variant of the *IL6R* gene may not be relevant in suicidal behavior; however, further studies are required to elucidate the whole participation of this gene or another hypothetic gene of the IL-6 pathway.

Previous studies have associated IL-6 with other mental disorders, particularly with depression [27]. Thus, IL-6 could participate in the morphological changes observed in the prefrontal cortex of individuals with an early stage of major depressive disorder [51]. Additionally, it has been described that increased levels of IL-6 predict depressive symptoms at 5 years of follow-up, regardless of BMI, sex, smoking, and age of the participants [52]. The variability and severity of depressive symptoms can predict suicide attempts in individuals at high risk [53,54]; however, due to the objectives of our study (to assess suicide attempts, lethality, and serum IL-6 level), we did not assess depression in our groups, so we consider this as a limitation of the study that should be considered when designing future studies.

Our study has some limitations: we observed a higher incidence of women attempting suicide than men. These results are consistent with previous reports in the literature and with the suicide paradox, where it has been described that women attempt suicide up to three times more than men; however, men die by suicide up to two times more than women [55,56,57,58]. It is important to emphasize that we focused on studying suicide attempts and not completed suicide.

The majority of individuals included in the comparison group were men recruited from the blood banks of the participating hospitals. Although it has been described that more women donate blood compared to men [59], it is possible that, in our sample, the type of study or the invitation to participate had a psychological impact on women, so men were more likely to accept the invitation.

The number of individuals included in the comparison group was higher than in the cases group; this because the number of individuals who attempted suicide was, fortunately, much smaller than the number of individuals who did not attempt suicide.

Additionally, the age of individuals in the suicide attempt group was younger than in the comparison group. It has been described that women have an earlier age of onset of suicide attempts compared to men [60]. Women being the majority in the suicide attempts group, it explains the differences of age in our study. Finally, the cross-sectional design of the study did not allow causality to be determined.

This study also has some strengths: it is the first study conducted on the Mexican population that evaluates the IL-6 levels in patients with suicidal behavior and that contemplates the level of lethality.

We believe that studies such as ours could open new areas of research with cytokines (such as IL-6) and some genetic variants. Or any other molecule that confers a predisposition to the development of mental disorders, particularly suicide attempts. Studying associated molecules/pathways in future studies could lead to the implementation of strategies focused on prevention, target treatments, and the search for future biomarkers.

## 5. Conclusions

We found increased levels of IL-6 serum in individuals who had just attempted suicide. Nonetheless, we did not observe an association between the IL-6 levels and the lethality of suicide attempts. Additionally, we observed no association between the rs2228145 polymorphism of the IL-6R gene and individuals with suicide attempts.

Therefore, we suggest that the serum levels of IL-6 in individuals with suicide attempts should be considered in future research aimed at finding biomarkers in these individuals in order to create fundamental prevention strategies and reduce suicide mortality.

Our results need to be replicated in other populations and larger samples.

## Figures and Tables

**Table 1:** Demographic characteristics of individuals with suicide attempts and the control group.

Characteristic	Total Sample *n* (%) *n* = 84	Comparison Group *n* (%) *n* = 66	Suicide Attempt Group *n* (%) *n* = 18	Statistics
**Sociodemographic characteristics**				
Sex				
Men	49 (58.3)	45 (68.2)	4 (22.2)	***x*^2^ = 12.29, *p* = 0.001**
Women	35 (41.7)	21 (31.8)	14 (77.8)	
Marital Status				
Single	36 (42.9)	27 (40.9)	9 (50)	*x*^2^ = 0.49, *p* = 0.78
Married	42 (50.0)	34 (51.5)	8 (44.4)	
Widower	6 (7.1)	5 (7.6)	1 (5.6)	
Occupation				
Unemployed	17 (20.2)	14 (21.2)	3 (16.7)	*x*^2^ = 2.01, *p* = 0.73
Homemaker	26 (31.0)	19 (28.8)	7 (38.9)	
Student	2 (2.3)	1 (1.5)	1 (5.5)	
Part-time employed	14 (16.7)	11 (16.7)	3 (16.7)	
Full time employed	25 (29.8)	21 (31.8)	4 (22.2)	
Age	42.19 ± 12.08	43.71 ± 11.83	36.63 ± 11.64	***t* = 2.25, *p* = 0.02**
Years of schooling	9.79 ± 4.17	9.34 ± 3.83	11.16 ± 5.12	*t* = −1.65, *p* = 0.10
**Clinical characteristics**				
Alcohol consumption				
Yes	37 (44.0)	30 (45.5)	7 (38.9)	*x*^2^ = 0.24, *p* = 0.79
No	47 (56.0)	36 (54.5)	11 (61.1)	
Cigarette Smoking				
Yes	10 (11.9)	7 (10.6)	3 (16.7)	*x*^2^ = 0.49, *p* = 0.36
No	74 (88.1)	59 (89.4)	15 (83.3)	
**Anthropometric Measurements**				
Weight (Kg)	80.01 ± 16.34	81.45 ± 16.71	74.72 ± 14.07	*t* = −1.72, *p* = 0.09
Height (cm)	163.14 ± 9.78	158.61 ± 6.92	164.37 ± 10.12	***t* = −2.81, *p* = 0.008**
Body Mass Index (kg/m^2^)	29.93 ± 4.90	30.02 ± 4.89	29.61 ± 5.03	*t* = −0.30, *p* = 0.76

Data are expressed as the mean ± standard deviation or *n* and percentage. Numbers in bold show significant statistical difference.

**Table 2:** Genotype and allele distribution of *IL-6R* rs2228145 in the suicide attempts and the comparison group.

rs2228145 Polymorphism						
Genotype/Allele	Comparison Group (*n* = 66)		Suicide Attempt (*n* = 18)		*x* ^2^	*p*
Genotype	*n*	%	*n*	%		
*AA*	14	21.2	5	27.8	3.638	0.16
*AC*	44	66.7	8	44.4		
*CC*	8	12.1	5	27.8		
**Allele**						
*A*	72	54.5	18	50	0.23	0.62
*C*	60	45.5	18	50		

## Data Availability

Not applicable.
